# 4-(Benz­yloxy)benzaldehyde

**DOI:** 10.1107/S1600536810027200

**Published:** 2010-07-24

**Authors:** Alan R. Kennedy, Zaccheus R. Kipkorir, Claire I. Muhanji, Maurice O. Okoth

**Affiliations:** a Department of Pure & Applied Chemistry, University of Strathclyde, 295 Cathedral Street, Glasgow G1 1XL, Scotland; b Department of Chemistry and Biochemistry, Moi University, PO Box 1125-30100, Eldoret, Kenya

## Abstract

The title compound, C_14_H_12_O_2_, has an essentially planar conformation with the two aromatic rings forming a dihedral angle of 5.23 (9)° and the aldehyde group lying in the plane of its aromatic group [maximum deviation = 0.204 (3) Å]. Weak inter­molecular C—H⋯O contacts are found to be shortest between the aldehyde O-atom acceptor and the H atoms of the methyl­ene group.

## Related literature

For discussion of C—H⋯O contacts in a related meth­oxy derivative, see: Gerkin (1999 ▶). For other related structures, see: Allwood *et al.* (1985 ▶); Li & Chen (2008 ▶); Liu *et al.* (2006 ▶, 2007 ▶); Zhen *et al.* (2006 ▶). For background to the anti­retroviral treatment programme of AIDS, see: UNAIDS/WHO (2009 ▶). The established non-nucleoside reverse transcriptase inhibitors (NNRTIs) are susceptible to the development of viral resistance, emanating from mutations of amino acids in RT enzymes (Jones *et al.*, 2006 ▶). For the need for new small mol­ecules that target HIV-1 binding sites, see: Christer *et al.* (1998 ▶); Himmel *et al.* (2006 ▶). For related literature on our work in this area, see: Hunter *et al.* (2007 ▶); Muhanji (2006 ▶).
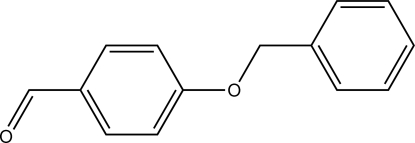


## Experimental

### 

#### Crystal data

C_14_H_12_O_2_
*M* *_r_* = 212.24
Orthorhombic, 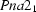

*a* = 11.4772 (11) Å
*b* = 12.9996 (12) Å
*c* = 7.2032 (6) Å
*V* = 1074.71 (17) Å^3^
*Z* = 4
Mo *K*α radiation
μ = 0.09 mm^−1^
*T* = 123 K
0.42 × 0.20 × 0.14 mm

#### Data collection

Oxford Diffraction Gemini S diffractometer
8432 measured reflections
1579 independent reflections
1130 reflections with *I* > 2σ(*I*)
*R*_int_ = 0.049

#### Refinement

*R*[*F* ^2^ > 2σ(*F* ^2^)] = 0.039
*wR*(*F* ^2^) = 0.071
*S* = 0.91
1579 reflections
150 parameters
1 restraint
H atoms treated by a mixture of independent and constrained refinement
Δρ_max_ = 0.18 e Å^−3^
Δρ_min_ = −0.17 e Å^−3^

### 

Data collection: *CrysAlis CCD* (Oxford Diffraction, 2007 ▶); cell refinement: *CrysAlis CCD*; data reduction: *CrysAlis RED* (Oxford Diffraction, 2007 ▶); program(s) used to solve structure: *SHELXS97* (Sheldrick, 2008 ▶); program(s) used to refine structure: *SHELXL97* (Sheldrick, 2008 ▶); molecular graphics: *ORTEP-3* (Farrugia, 1997 ▶); software used to prepare material for publication: *SHELXL97*.

## Supplementary Material

Crystal structure: contains datablocks global, I. DOI: 10.1107/S1600536810027200/gw2083sup1.cif

Structure factors: contains datablocks I. DOI: 10.1107/S1600536810027200/gw2083Isup2.hkl

Additional supplementary materials:  crystallographic information; 3D view; checkCIF report

## Figures and Tables

**Table 1:** Hydrogen-bond geometry (Å, °)

*D*—H⋯*A*	*D*—H	H⋯*A*	*D*⋯*A*	*D*—H⋯*A*
C1—H1*A*⋯O2^i^	0.99	2.50	3.324 (2)	141
C1—H1*B*⋯O2^ii^	0.99	2.53	3.478 (2)	160

Symmetry codes: (i) 
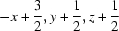
; (ii) 
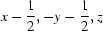
.

**Fig. 1.**
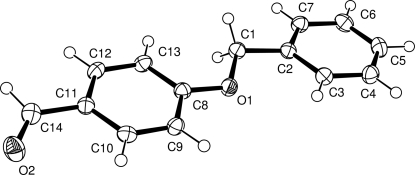
The molecular structure showing 50% probability displacement ellipsoids.

**Fig. 2.**
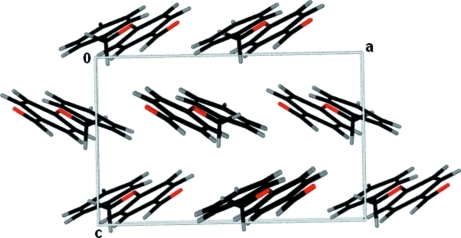
Packing diagram with view along the length of the b axis.

## supplementary crystallographic information

### Comment

A critical strategy for mitigating the impact of the Acquired Immunodeficiency Syndrome (AIDS) epidemic is the antiretroviral treatment programme (UNAIDS/WHO, 2009). The established non-nucleoside reverse transcriptase inhibitors (NNRTIs) are susceptible to the development of viral resistance, emanating from mutations of amino acids in RT enzymes (Jones *et al.*, 2006). The effectiveness of the drugs that have already been developed is thus affected by the emergence of drug resistant strains. Consequently, NNRTIs having a good activity against wild-type RT and the most prevalent mutant viral strains are needed. There is therefore the need to look for new compounds that are highly potent and less susceptible to mutations of RT- enzyme (Christer *et al.*, 1998). According to Himmel *et al.* (2006), new lead compounds that target novel binding sites are needed because of rapid emergence of these drug resistant variants of HIV-1 which has limited the efficacy of AIDS treatment. This study was therefore limited to the use of Wiener's topological index, a theoretical approach used in theoretical chemistry to predict the anti-HIV activity of phenylethylthiazolylthiourea (PETT) analogues.

The title compound, 4-(benzyloxy)benzaldehyde, was an intermediate in the production of such target compounds. It was found to exist as discrete molecules (Figure 1), although there are some non-classical hydrogen bonding C—H···O interactions involving the aldehyde O atom and both the methylene H atoms (H···O 2.50 and 2.53 Å) and aromatic H atoms (2.69 and 2.80 Å). Similar interactions are described for the similar 2-methoxy vanillin derivitive by Gerkin (1999). All contacts to the ether O atom are longer than these.

Bond lengths are similar to those found in the structures of related compounds and the aldehyde is coplanar with the ring in all cases (here C10C11C14O2 = -6.3 (3) °. However, two different conformations are found for these compounds. In common with three other derivatives (Li & Chen (2008); Liu *et al.* (2006); Zhen *et al.* (2006)), the two aromatic rings of 4-(benzyloxy)benzaldehyde approach coplanarity (C13C8C2C7 = -9.2 (3)°), whilst the similarly substituted species described by Gerkin (1999), Allwood *et al.* (1985) and Liu *et al.* (2007) are very twisted (torsion angle range 31.7 to 99.1 °).

### Experimental

All reactions in the preparation of 4-(benzyloxy)benzaldehyde were performed under an atmosphere of nitrogen gas. 5.0 g of 4-hydroxybenzaldehyde (40.98 mmol), 5.0 ml of benzylbromide (42.05 mmol) and 20.0 g of anhydrous potassium carbonate (144.27 mmol) in ethanol were refluxed for 14 hours. Potassium carbonate was filtered out and large volumes of EtOAc were used to wash the residue. Rotavapour apparatus was used to remove the solvent. The residual mass was dissolved in 50 ml Et_2_O. Two portions of 50 mL saturated sodium chloride solution were used to wash the Et_2_O solution. Thereafter, it was washed with one portion of 5% sodium hydroxide solution. Finally, the Et_2_O solution was washed with distilled water. Anhydrous magnesium sulphate was used to dry the Et_2_O solution and the solvent removed under reduced pressure. The crude product was then recrystallized from ethanol to give colorless crystals (7.58 g, 87.4%). Mp: 338-339 K.

### Refinement

The aldehyde H atom (H14) was refined freely, but all other atoms were placed in calculated positions and refined in riding modes with U_iso_(H) = 1.2U_eq_(C). C—H distances 0.95 and 0.99 Å for CH and CH_2_ respectively.

### Crystal data

**Table:**

C_14_H_12_O_2_	*D*_x_ = 1.312 Mg m^−^^3^
*M**_r_* = 212.24	Mo *K*α radiation, λ = 0.71073 Å
Orthorhombic, *P**n**a*2_1_	Cell parameters from 2444 reflections
*a* = 11.4772 (11) Å	θ = 2.8–29.9°
*b* = 12.9996 (12) Å	µ = 0.09 mm^−^^1^
*c* = 7.2032 (6) Å	*T* = 123 K
*V* = 1074.71 (17) Å^3^	Block, colourless
*Z* = 4	0.42 × 0.20 × 0.14 mm
*F*(000) = 448	

### Data collection

**Table:**

Oxford Diffraction Gemini S diffractometer	*R*_int_ = 0.049
Radiation source: fine-focus sealed tube	θ_max_ = 30.0°, θ_min_ = 3.1°
graphite	*h* = −15→15
ω scans	*k* = −18→13
8432 measured reflections	*l* = −9→9
1579 independent reflections	3 standard reflections every 240 min
1130 reflections with *I* > 2σ(*I*)	intensity decay: none

### Refinement

**Table:**

Refinement on *F*^2^	Secondary atom site location: difference Fourier map
Least-squares matrix: full	Hydrogen site location: inferred from neighbouring sites
*R*[*F*^2^ > 2σ(*F*^2^)] = 0.039	H atoms treated by a mixture of independent and constrained refinement
*wR*(*F*^2^) = 0.071	*w* = 1/[σ^2^(*F*_o_^2^) + (0.0342*P*)^2^] where *P* = (*F*_o_^2^ + 2*F*_c_^2^)/3
*S* = 0.91	(Δ/σ)_max_ < 0.001
1579 reflections	Δρ_max_ = 0.18 e Å^−^^3^
150 parameters	Δρ_min_ = −0.17 e Å^−^^3^
1 restraint	Extinction correction: *SHELXL97* (Sheldrick, 2008), Fc^*^=kFc[1+0.001xFc^2^λ^3^/sin(2θ)]^-1/4^
Primary atom site location: structure-invariant direct methods	Extinction coefficient: 0.0052 (11)

### Special details

**Table:**

Geometry. All esds (except the esd in the dihedral angle between two l.s. planes) are estimated using the full covariance matrix. The cell esds are taken into account individually in the estimation of esds in distances, angles and torsion angles; correlations between esds in cell parameters are only used when they are defined by crystal symmetry. An approximate (isotropic) treatment of cell esds is used for estimating esds involving l.s. planes.
Refinement. Refinement of *F*^2^ against ALL reflections. The weighted *R*-factor wR and goodness of fit *S* are based on *F*^2^, conventional *R*-factors *R* are based on *F*, with *F* set to zero for negative *F*^2^. The threshold expression of *F*^2^ > σ(*F*^2^) is used only for calculating *R*-factors(gt) etc. and is not relevant to the choice of reflections for refinement. *R*-factors based on *F*^2^ are statistically about twice as large as those based on *F*, and *R*- factors based on ALL data will be even larger.

### Fractional atomic coordinates and isotropic or equivalent isotropic displacement parameters (Å^2^)

**Table:**

	*x*	*y*	*z*	*U*_iso_*/*U*_eq_	
O1	0.63724 (11)	0.04432 (8)	0.81224 (19)	0.0256 (3)	
O2	0.81066 (12)	−0.42284 (9)	0.8002 (2)	0.0360 (4)	
C1	0.53896 (16)	0.08862 (13)	0.9065 (2)	0.0232 (4)	
H1A	0.5446	0.0748	1.0413	0.028*	
H1B	0.4660	0.0573	0.8599	0.028*	
C2	0.53725 (17)	0.20292 (13)	0.8725 (2)	0.0212 (4)	
C3	0.62873 (17)	0.25573 (12)	0.7892 (3)	0.0235 (4)	
H3	0.6963	0.2195	0.7503	0.028*	
C4	0.62149 (18)	0.36164 (13)	0.7626 (3)	0.0276 (5)	
H4	0.6838	0.3974	0.7045	0.033*	
C5	0.52362 (18)	0.41501 (14)	0.8205 (3)	0.0294 (5)	
H5	0.5186	0.4872	0.8019	0.035*	
C6	0.43353 (18)	0.36301 (14)	0.9054 (3)	0.0293 (5)	
H6	0.3670	0.3998	0.9469	0.035*	
C7	0.43932 (18)	0.25775 (14)	0.9304 (2)	0.0251 (4)	
H7	0.3762	0.2225	0.9874	0.030*	
C8	0.65609 (17)	−0.05854 (13)	0.8365 (3)	0.0219 (4)	
C9	0.75866 (17)	−0.09649 (14)	0.7567 (2)	0.0231 (4)	
H9	0.8097	−0.0512	0.6926	0.028*	
C10	0.78550 (16)	−0.19922 (13)	0.7712 (3)	0.0232 (4)	
H10	0.8551	−0.2250	0.7170	0.028*	
C11	0.71047 (16)	−0.26592 (13)	0.8657 (2)	0.0211 (4)	
C12	0.60968 (17)	−0.22681 (14)	0.9451 (2)	0.0234 (4)	
H12	0.5590	−0.2720	1.0102	0.028*	
C13	0.58121 (17)	−0.12411 (13)	0.9319 (2)	0.0236 (4)	
H13	0.5118	−0.0985	0.9869	0.028*	
C14	0.73311 (19)	−0.37705 (14)	0.8791 (3)	0.0271 (5)	
H14	0.6736 (19)	−0.4158 (13)	0.961 (3)	0.033 (6)*	

### Atomic displacement parameters (Å^2^)

**Table:**

	*U*^11^	*U*^22^	*U*^33^	*U*^12^	*U*^13^	*U*^23^
O1	0.0262 (7)	0.0207 (6)	0.0301 (8)	0.0024 (6)	0.0083 (6)	0.0016 (6)
O2	0.0320 (9)	0.0287 (8)	0.0474 (9)	0.0057 (6)	0.0023 (8)	−0.0033 (7)
C1	0.0216 (11)	0.0224 (10)	0.0255 (11)	0.0029 (8)	0.0033 (8)	0.0005 (8)
C2	0.0210 (10)	0.0224 (10)	0.0201 (10)	−0.0017 (8)	−0.0036 (8)	−0.0005 (7)
C3	0.0202 (10)	0.0248 (10)	0.0256 (11)	0.0000 (8)	0.0003 (8)	−0.0017 (9)
C4	0.0260 (12)	0.0279 (11)	0.0288 (12)	−0.0055 (8)	0.0000 (9)	0.0008 (9)
C5	0.0339 (12)	0.0203 (10)	0.0340 (11)	0.0003 (9)	−0.0044 (10)	−0.0012 (9)
C6	0.0274 (12)	0.0288 (10)	0.0316 (12)	0.0049 (9)	−0.0009 (9)	−0.0058 (9)
C7	0.0224 (11)	0.0275 (11)	0.0254 (10)	−0.0003 (8)	0.0027 (9)	−0.0002 (8)
C8	0.0234 (10)	0.0224 (10)	0.0199 (10)	0.0006 (8)	−0.0008 (8)	−0.0002 (8)
C9	0.0211 (11)	0.0243 (10)	0.0239 (10)	−0.0043 (8)	0.0033 (8)	0.0010 (8)
C10	0.0212 (10)	0.0270 (10)	0.0213 (10)	0.0006 (8)	0.0014 (8)	−0.0027 (8)
C11	0.0222 (10)	0.0216 (10)	0.0195 (9)	−0.0009 (8)	−0.0034 (8)	0.0004 (8)
C12	0.0230 (11)	0.0254 (10)	0.0217 (10)	−0.0045 (9)	0.0012 (8)	0.0044 (8)
C13	0.0217 (11)	0.0266 (10)	0.0225 (10)	0.0014 (8)	0.0028 (8)	0.0024 (8)
C14	0.0261 (12)	0.0261 (11)	0.0290 (11)	−0.0021 (9)	−0.0046 (9)	0.0009 (9)

### Geometric parameters (Å, °)

**Table:**

O1—C8	1.3657 (18)	C6—H6	0.9500
O1—C1	1.437 (2)	C7—H7	0.9500
O2—C14	1.212 (2)	C8—C13	1.392 (3)
C1—C2	1.506 (2)	C8—C9	1.400 (2)
C1—H1A	0.9900	C9—C10	1.375 (2)
C1—H1B	0.9900	C9—H9	0.9500
C2—C3	1.391 (3)	C10—C11	1.399 (2)
C2—C7	1.395 (3)	C10—H10	0.9500
C3—C4	1.393 (2)	C11—C12	1.387 (2)
C3—H3	0.9500	C11—C14	1.471 (3)
C4—C5	1.385 (3)	C12—C13	1.378 (2)
C4—H4	0.9500	C12—H12	0.9500
C5—C6	1.378 (3)	C13—H13	0.9500
C5—H5	0.9500	C14—H14	1.03 (2)
C6—C7	1.382 (3)		
			
C8—O1—C1	117.15 (13)	C6—C7—H7	119.8
O1—C1—C2	109.20 (14)	C2—C7—H7	119.8
O1—C1—H1A	109.8	O1—C8—C13	124.39 (17)
C2—C1—H1A	109.8	O1—C8—C9	115.20 (16)
O1—C1—H1B	109.8	C13—C8—C9	120.41 (16)
C2—C1—H1B	109.8	C10—C9—C8	119.99 (16)
H1A—C1—H1B	108.3	C10—C9—H9	120.0
C3—C2—C7	119.04 (16)	C8—C9—H9	120.0
C3—C2—C1	123.18 (16)	C9—C10—C11	120.08 (17)
C7—C2—C1	117.77 (16)	C9—C10—H10	120.0
C2—C3—C4	120.15 (18)	C11—C10—H10	120.0
C2—C3—H3	119.9	C12—C11—C10	119.13 (16)
C4—C3—H3	119.9	C12—C11—C14	118.69 (16)
C5—C4—C3	120.16 (18)	C10—C11—C14	122.15 (17)
C5—C4—H4	119.9	C13—C12—C11	121.64 (16)
C3—C4—H4	119.9	C13—C12—H12	119.2
C6—C5—C4	119.76 (17)	C11—C12—H12	119.2
C6—C5—H5	120.1	C12—C13—C8	118.75 (17)
C4—C5—H5	120.1	C12—C13—H13	120.6
C5—C6—C7	120.50 (19)	C8—C13—H13	120.6
C5—C6—H6	119.7	O2—C14—C11	125.5 (2)
C7—C6—H6	119.7	O2—C14—H14	120.9 (10)
C6—C7—C2	120.38 (18)	C11—C14—H14	113.5 (10)
			
C8—O1—C1—C2	176.49 (14)	O1—C8—C9—C10	−179.33 (17)
O1—C1—C2—C3	−9.8 (2)	C13—C8—C9—C10	0.5 (3)
O1—C1—C2—C7	171.08 (16)	C8—C9—C10—C11	0.0 (3)
C7—C2—C3—C4	−0.7 (3)	C9—C10—C11—C12	−0.5 (3)
C1—C2—C3—C4	−179.76 (16)	C9—C10—C11—C14	177.71 (18)
C2—C3—C4—C5	0.6 (3)	C10—C11—C12—C13	0.5 (3)
C3—C4—C5—C6	0.3 (3)	C14—C11—C12—C13	−177.70 (17)
C4—C5—C6—C7	−1.0 (3)	C11—C12—C13—C8	−0.1 (3)
C5—C6—C7—C2	0.9 (3)	O1—C8—C13—C12	179.39 (17)
C3—C2—C7—C6	−0.1 (3)	C9—C8—C13—C12	−0.4 (3)
C1—C2—C7—C6	179.06 (16)	C12—C11—C14—O2	171.8 (2)
C1—O1—C8—C13	6.0 (2)	C10—C11—C14—O2	−6.3 (3)
C1—O1—C8—C9	−174.23 (17)		

### Hydrogen-bond geometry (Å, °)

**Table:**

*D*—H···*A*	*D*—H	H···*A*	*D*···*A*	*D*—H···*A*
C1—H1A···O2^i^	0.99	2.50	3.324 (2)	141
C1—H1B···O2^ii^	0.99	2.53	3.478 (2)	160

Symmetry codes: (i) −*x*+3/2, *y*+1/2, *z*+1/2; (ii) *x*−1/2, −*y*−1/2, *z*.
